# Symptom dimensions of anxiety in Parkinson’s disease: Replication study in a neuropsychiatric patient population

**DOI:** 10.1016/j.prdoa.2021.100117

**Published:** 2021-11-10

**Authors:** Sonja Rutten, Erwin E.H. van Wegen, Ires Ghielen, Bridget Schoon, Odile A. van den Heuvel

**Affiliations:** a Amsterdam University Medical Centers (Amsterdam UMC), Vrije Universiteit Amsterdam, Department of Psychiatry, Amsterdam Neuroscience, De Boelelaan 1117, 1081 HV Amsterdam, Netherlands; b Amsterdam UMC, Vrije Universiteit Amsterdam, Department of Anatomy & Neurosciences, Amsterdam Neuroscience, De Boelelaan 1108, 1081 HZ Amsterdam, Netherlands; c Amsterdam UMC, Vrije Universiteit Amsterdam, Department of Rehabilitation Medicine, Amsterdam Movement Sciences, Amsterdam Neuroscience, De Boelelaan 1117, 1081 HV Amsterdam, Netherlands

**Keywords:** Parkinson’s disease, Anxiety, Factor analysis, Replication, Neuropsychiatry, Symptom interaction, Non-motor symptoms

## Abstract

**Introduction:**

Anxiety disorders occur in approximately one third of people with Parkinson’s disease (PD), and have a major impact on patient and caregiver wellbeing. In order to better understand and diagnose anxiety in PD patients, we investigated the generalizability of the results of a previous factor analysis on anxiety symptoms to a sample of PD patients with neuropsychiatric symptoms.

**Methods:**

In this cross-sectional study, anxiety symptoms were measured with the Beck Anxiety Inventory (BAI) in 123 PD patients who were referred for neuropsychiatric diagnostics and treatment. Subscales of anxiety were analyzed through principal component analysis of BAI items. The associations between BAI subscales and symptoms of motor and cognitive function and depression were assessed through regression analyses.

**Results:**

Similar to the previous factor analysis, we found one psychological (affective) and four somatic subscales of anxiety in the BAI. The affective subscale was the principal component explaining 35.9% of the variance. The scores on the total BAI and the affective subscale were significantly associated with depressive symptoms. In a post-hoc analysis, the affective subscale had equal power as compared to the total BAI in predicting whether or not participants were diagnosed with an anxiety disorder after psychiatric evaluation.

**Conclusion:**

In this study, we replicated our previous findings of one affective and multiple somatic subscales of the BAI. The 7-item affective subscale of the BAI shows potential as a screening tool for non-episodic anxiety in PD. However, in clinical practice, we recommend evaluating anxiety symptoms in the context of other PD symptoms, including motor, autonomic, and other (neuro)psychiatric symptoms.

## Highlights

• We replicated a previous factor analysis on anxiety in PD.
• The BAI consists of one psychological and several somatic subscales in PD.
• The affective subscale of the BAI has equal predictive power in diagnosing anxiety.
• Anxiety symptoms need to be evaluated in the context of other PD symptoms.

## 1 Introduction

Anxiety disorders are present in an estimated 31% of patients with Parkinson’s Disease (PD), with generalized anxiety disorder being the most common [1], [2]. Around 45% of PD patients suffer from clinically relevant anxiety [3]. Both from clinical experience and previous research, anxiety can be linked to fluctuations in available dopamine related to timing of PD medication and is often comorbid or secondary to depression, psychosis, and cognitive decline [3], [4], [5]. Anxiety is therefore one of the most prevalent neuropsychiatric features in PD patients and can be very debilitating. In addition, its presence is one of the most significant predictors of health-related quality of life in PD [6], [7], and greatly impacts caregiver burden [8]. Anxiety can worsen motor symptoms, such as tremor and freezing [9], [10] and can create a vicious cycle, in which motor symptoms trigger anxiety or distress, which can in turn exacerbate motor symptoms. These reciprocal interactions between motor and non-motor symptoms are not only seen in clinical practice but are supported by scientific research [11], [12], [13]. Despite its large impact on patient well-being and caregiver burden, anxiety in PD is still poorly understood and evidence on the treatment of anxiety in PD patients is limited [14], [15].

Self-report questionnaires, such as the Beck Anxiety Inventory (BAI), can be used to quantify anxiety symptoms [16]. However, diagnosing anxiety is complicated by its overlap and reciprocity with motor (e.g., tremor, rigidity, freezing) and autonomic symptoms (e.g., excessive perspiration) [12], [17]. The interpretation of the total score on self-report questionnaires can therefore be complicated. Evaluation of the specific symptom dimensions (or subscales of a questionnaire) can be a useful approach [18]. One statistical technique for extracting these subscales is principal components analysis (PCA). In a previous study in PD patients [19], a factor solution of the BAI was not found by using PCA, which might be explained by the great heterogeneity of the investigated study population. Another study investigated the dimensionality of the BAI by principal axis factoring, and found five factors, including one distinct PD motor subscale [12]. In a previously conducted PCA by our research group, based on a large cohort of patients that were referred to our outpatient clinic for movement disorders, we found that the BAI encompasses one affective and four somatic factors [18]. A significant association between the symptoms of anxiety and depression was found, and severity of motor symptoms showed significant associations with the somatic factors of the BAI, and not with the affective subscale.

In the current study we aim to replicate the findings of Rutten et al. (2015) in an independent sample of PD patients who were specifically referred for specialist neuropsychiatric evaluation [18]. Using the same methodology in a different patient sample enables us to investigate the generalizability of the previous findings [20].

## 2 Methods

### 2.1 Subjects

The data used in this cross-sectional study were routinely collected at the Center for Neuropsychiatry in Parkinson’s disease (CNP) of the Amsterdam University medical center, location VUmc in Amsterdam, the Netherlands. The CNP is a specialized outpatient department for the diagnosis and treatment of PD patients experiencing neuropsychiatric symptoms. Patients are referred to the CNP by neurologists, general practitioners and specialists in geriatric medicine. Data of 176 patients were collected between April 2014 and February 2018. Patients were included if they were previously diagnosed with idiopathic Parkinson’s disease. All study participants provided written informed consent for their clinical data to be used in scientific research. Since several participants were included in both the previous and the current databases, we excluded overlapping patient data collected within five years after the data collection of Rutten et al. (2015) [18].

### 2.2 Assessments

Severity of symptoms of anxiety was measured with the BAI [16]. The BAI is a self-report instrument that consists of 21 items, enabling patients to report the symptoms of anxiety experienced in the previous week on a 4-point Likert scale, ranging from 0 (not at all) to 3 (severely). The total score can range from 0 to 63. The cut-off score used for clinically relevant anxiety in patients with PD is >12 [19].

The Montreal Cognitive Assessment (MoCA) was used to screen the patient group for possible cognitive decline [21]. The United Parkinson Disease Rating Scale-III (UPDRS-III) score was used to assess the severity of motor symptoms [22]. The Beck Depression Inventory (BDI) was used to examine self-reported symptoms of depression [23]. The BDI cut-off score for clinically relevant symptoms of depression is 14 [24]. Moreover, sex, age, disease duration, and the use of dopaminergic medication (0 = no, 1 = yes) were recorded for study participants.

Finally, we recorded the patient’s psychiatric history as well as the psychiatric diagnoses given after clinical evaluation by the assessing psychiatrists (OvdH/SR). Due to the time-frame in which data was collected, both the Diagnostic and Statistical Manual of Mental Disorders (DSM-)IV and the DSM-5 were used [25].

### 2.3 Statistical analyses

All analyses were performed using SPSS Statistics 22 for Windows with a two-sided significance level of *p* < 0.05. The acceptability of missing values of the BAI and BDI was set to less than three missing items, i.e. 16.67%. In case of more missing data, the patient was excluded from further analysis. Mean imputation was used for residual missing data of the BAI and BDI. Data were excluded pairwise when the total MoCA score or UPDRS-III score was not available since imputation was not considered to be reliable.

PCA was used to assess the dimensionality of the BAI. In PCA, items that share the most common explained variance cluster together in factors. In order to determine the number of factors that can be reliably extracted, the ‘scree plot’ criterion and the Guttman-Kaiser Eigenvalue greater-than-one rule were used. Oblimin rotation was used since it was expected that the different factors correlate with each other. The resulting factors of the BAI can be considered subscales of the BAI.

The associations between the BAI and its subscales, and the BDI, MoCA, and UPDRS-III were investigated by conducting multiple linear regression analyses. Assumptions of normality and homoscedasticity of residuals were checked. Multicollinearity was evaluated by calculating the variance inflation factor (VIF) and investigation of the correlation matrix. In the first set of regression analyses, the total BAI score was the dependent variable. In the second set, the scores on the subscales of the BAI derived from the PCA were the dependent variables. The independent variables were the total score on the BDI, UPDRS-III, and MoCA. First, we investigated the association between the dependent and independent variables in an unadjusted model. Next, we adjusted the model for age, gender, use of dopaminergic medication, and the two other independent variables of interest, i.e. the BDI, MoCA, and UPDRS-III.

## 3 Results

Of the available sample of 176 patients, 48 patients were excluded due to missing data. Of the remaining 128 patients, five were excluded due to overlap with the sample of Rutten et al. (2015) [18], resulting in a total sample of 123 participants.

Demographic and clinical characteristics of the participants are shown in Table 1. In 14 participants, data on the UPDRS-III was missing, and the disease duration was unknown in 38 participants. The majority of participants was male, mean age was 66.1 years.

**Table 1:** Clinical and demographic characteristics (N = 123).

	Mean	SD	Range
% Female	38.2		
Age	66.1	9.8	34–86
Disease duration (years) (n = 85)	8.4	6.9	0–29
MoCA score	23.8	4.7	5–30
UPDRS-III score (n = 109)	27.3	14.8	1–76
Total BAI score	20.3	11.4	0–55
Total BDI score	18.2	9.3	1–47
% Use dopaminergic medication	93.5		
% Treatment of psychiatric symptoms prior to PD diagnosis	49.6		

The mean BAI total score of all participants was 20.3 (±11.4), and 77.4% of the participants had a BAI score higher than 12, indicating clinically relevant symptoms of anxiety.

The vast majority of the participants (91.9%) received at least one psychiatric diagnosis after psychiatric evaluation (see Table 2). Sixty-five participants (52.9% of the total sample) met DSM-IV or -5 criteria for an anxiety disorder. Of these 65 participants, 79.3% had a least one comorbid psychiatric diagnosis. Depressive disorders were the most frequent occurring comorbidity, as they were diagnosed in 42.3% of participants with an anxiety disorder.

**Table 2:** Psychiatric diagnoses as given by assessing psychiatrist (N = 123)

Diagnosis given (DSM-IV or -5)	%
**No diagnosis**	8.1
**Anxiety disorders**	52.9
Due to somatic disorder (PD)	41.5
Panic disorder	6.5
Generalized anxiety disorder	3.3
Social anxiety disorder	1.6
**Depressive disorders**	41.4
Due to somatic disorder (PD)	25.2
Persistent depressive disorder (dysthymia)	0.8
Major depressive disorder	15.4
**Neurocognitive disorder**	31.7
Other specified disruptive, impulse-control and conduct disorder*	16.3
**Psychotic disorder**	12.9
**Sleep-wake disorder**	15.4

* all patients fulfilling criteria for this DSM category had an impulse control disorder.

Half of the participants (49.6%) had sought treatment for psychiatric symptoms prior to receiving a PD diagnosis. Of this sub-population, 45.5% did so specifically for symptoms of anxiety.

### 3.1 Dimensions of the BAI

Examination of the scree-plot and the Eigenvalue greater-than-one-rule suggested five factors. All BAI-items had a loading greater than 0.4 on at least one of the factors and therefore all items were included in the factor solution. No items had a loading greater than 0.4 on multiple factors.

The five extracted factors were considered subscales, and were given the following labels: affective, thermoregulation, cardiopulmonary, unsteadiness and ‘undefined’. The last factor included three items: dizzy or lightheaded, indigestion and faint/lightheaded. The Eigenvalue and percentage of explained variance of this factor were lowest of all factors (1.160 and 5.5, respectively), and the three items of this subscale had relatively low factor loadings of 0.620 to 0.666. Moreover, in contrast to the other factors, we could not interpret this factor clinically as a specific symptom dimension of anxiety. Therefore, we decided not to label this factor as a subscale and excluded it from further analyses.

**Fig. 1:**
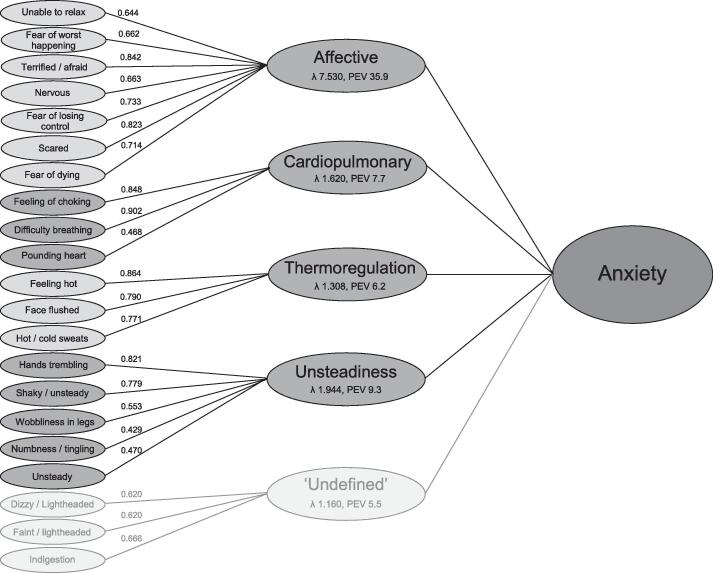
Graphical display of the results of the PCA of the BAI. Factor loadings, Eigenvalues (ʎ) and percentage of explained variance (PEV) are displayed.

The factor solution explained a total of 64.6% of the variance, 59.1% excluding the last factor. The affective subscale explained 35.9% of the total variance. Fig. 1 shows the distribution of the BAI items over the five subscales, with the corresponding factor loadings.

### 3.2 Associations between anxiety, depression, and cognitive and motor dysfunction

Histograms showed that the residuals of both the total BAI and its subscale scores had a positively skewed distribution. As normality did not improve after transformation of the data, we used the original data for the analyses to facilitate interpretability of results. Homoscedasticity and non-multicollinearity of the data was confirmed. The VIF ranged from 1.02 to 1.44. The correlation matrix is presented in supplementary Table S1.

Table 3 shows the results of the adjusted multiple regression analyses with the BAI total score and scores on the subscales of the BAI as dependent variables. The unadjusted model can be found in Supplementary Table S2.

**Table 3:** Results of multiple linear regression analyses of the BAI total score and score on subscales of the BAI with the BDI, MoCA and UPDRS-III, adjusted for gender, age and use of PD medication.

DEPENDENT VARIABLE → INDEPENDENT VARIABLE ↓	*Total BAI score*			*Affective*			*Thermoregulation*			*Cardiopulmonary*			*Unsteadiness*		
	B	95% CI of B	β	B	95% CI of B	β	B	95% CI of B	β	B	95% CI of B	β	B	95% CI of B	β
(constant)	11.915	−10.559 to 34.389		4.854	−4.602 to 14.310		3.154	−2.525 to 8.834		3.621	−0.955 to 8.197		2.992	−5.719 to 11.704	
BDI	0.691	0.503 to 0.880	0,569*	0.246	0.167 to 0.326	0.503*	0.008	−0.039 to 0.054	0.035	−0.009	−0.048 to 0.029	−0.046	0.004	−0.069 to 0.077	0.011
MoCA	−0.339	−0.786 to 0.108	−0.141	−0.177	−0.365 to −0.011	−0.182	−0.001	−0.114 to 0.112	−0.002	−0.045	−0.136 to 0.046	−0.117	0.069	−0.105 to 0.242	0.095
UPDRS-III	−0.003	−0.130 to 0.123	−0.004	−0.027	−0.080 to 0.026	−0.087	0.009	−0.023 to 0.041	0.058	−0.015	−0.041 to 0.010	−0.124	0.005	−0.045 to 0.054	0.019
Gender	2.729	−0.856 to 6.313	0.117	0.993	−0.515 to 2.501	0.106	0.049	−0.857 to 0.955	0.011	0.122	−0.607 to 0.852	0.033	−0.507	−1.896 to 0.882	−0.072
Age	0.032	−0.179 to 0.243	0.028	0.020	−0.069 to 0.108	0.042	−0.008	−0.062 to 0.045	−0.037	−0.024	−0.067 to 0.019	−0.131	0.023	−0.058 to 0.105	0.067
PD MEDICATION	−2.074	−9.182 to 5.034	−0.045	−0.493	−3.483 to 2.498	−0.027	−0.855	−2.651 to 0.941	−0.095	1.170	−0.277 to 2.618	0.158	0.018	−2.737 to 2.773	0.001
R^2^	0.402			0.349			0.013			0.047			0.012		

BAI = Beck Anxiety Inventory; BDI = Beck Depression Inventory; UPDRS-III = Unified Parkinson’s Disease Rating Scale – part three (motor examination); MoCA = Montreal Cognitive Assessment, B = regression coefficient, 95% CI of B = 95% confidence intervals of B, β = standardized regression coefficient.

* = p < 0.05.

The BAI total score and the affective subscale of the BAI were both significantly associated with the BDI score and MoCA score. In the final adjusted model, the associations between the MoCA score and BAI total and affective subscale scores were no longer statistically significant due to confounding by the BDI score. No other significant associations were found.

### 3.3 Post-hoc analysis

We conducted a post-hoc analysis to investigate whether the score on the affective subscale of the BAI alone was better in predicting an anxiety disorder diagnosis given by a psychiatrist compared to the total score of the BAI. In order to do so, we calculated the area under the Receiver Operating Characteristic (ROC) curve of both the BAI total score and the affective subscale of the BAI in relation to an anxiety disorder diagnosis (yes or no).

The BAI total score and the BAI affective subscale score showed similar power in predicting an anxiety disorder diagnosis given by a psychiatrist in this PD patient sample: the area under the ROC curve of the BAI total score was 0.77 (sd = 0.04, *p* < 0.001), and 0.75 (sd = 0.05, *p* < 0.001) for the affective subscale of the BAI.

## 4 Discussion

In this study, we replicated the findings of our previous study [18] in an independent PD patient sample with neuropsychiatric symptoms necessitating referral to an expert neuropsychiatric outpatient clinic.

From the data collected from the current CNP patient sample, PCA uncovered four clinically interpretable factors, representing subscales of the BAI, which we labeled as affective, thermoregulation, cardiopulmonary, and unsteadiness. Salazar et al. (2017) previously assessed dimensionality of the BAI in a population of 100 PD patients, using a different statistical approach, i.e. principal axis factoring, and found five factors [12]. As they were mainly interested in assessing the loading of BAI items that can be interpreted as motor symptoms of PD, they do not comment on the clinical interpretation of the other four factors. Their ‘motor’ factor and our ‘unsteadiness’ factor are quite similar: the BAI items ‘unsteadiness’, ‘wobbliness in legs’, ‘shaky/unsteady’ and ‘hands trembling’ load on both. However, there are few similarities between the other factors of both studies. This is probably due to the fact that they excluded patients with an anxiety disorder from their study population.

In the study of Leentjens et al. (2011), 34% of 342 included PD patients had a current DSM anxiety disorder [3]. Using PCA, they found two factors, that were clinically not interpretable. The authors of this study attribute this to the large heterogeneity of their study population. When comparing the clinical and demographic characteristics, the study sample of Leentjens and colleagues and our study sample are quite similar except for the level of anxiety: in our study population this was substantially higher (mean = 20.3, SD 11.4, as compared to mean = 12.6 SD 9.1).

When comparing our PCA to the results published by Rutten et al. (2015) [18], both studies show a partition of psychological and somatic symptom dimensions, and the highest percentage of the variance was explained by the affective subscale. This demonstrates the generalizability to an independent PD sample and confirms the robustness of our results. While the affective and thermoregulation subscales were exact replications, the cardiopulmonary and unsteadiness subscales showed a slightly different composition to the previously found subscales hypotension, hyperventilation, and trembling.

The BAI total score and the BAI affective subscale score were significantly associated with the BDI score. The association between the BAI and the BDI was also reported in the previous study [18]. This suggests a high co-occurrence of anxiety and depressive symptoms [5], as is also supported by Zhu and colleagues [4], who found that up to 70% of anxious PD patients also suffer from depression. The BAI total score and BAI affective subscale score were also significantly associated with the MoCA score. However, in the final adjusted model, this association was no longer significant due to confounding by the BDI score, explained by the high correlation between the BAI and BDI. In contrast to the previous study [18], no significant associations between somatic subscales and motor symptoms were found. The current patient sample showed more neuropsychiatric symptoms, but had a similar degree of motor dysfunction (see supplementary Table S3 for the independent samples t-tests results of the comparison of the sample characteristics). The slightly different distribution of BAI items over the somatic subscales might explain the absence of the associations with motor symptoms in the current sample.

Based on the BAI total score, 77.4% of the current patient sample showed clinically relevant anxiety. However, only 52.9% of participants received a formal DSM-IV or -5 anxiety diagnosis from the assessing psychiatrist. This could be due to symptom overlap and high co-occurrence with depression, in which case a depressive disorder might be more fitting with the clinical presentation in some cases. In addition, anxiety symptoms can occur in the context of other psychiatric disorders, such as psychosis, neurocognitive disorders, and dopamine dysregulation syndrome. Moreover, anxiety in PD patients often has an atypical presentation, and does not fit DSM criteria for a specific anxiety disorders [26]. Another possible explanation is that the severity of anxiety may be overestimated by the BAI [17], since motor and autonomic symptoms of PD could have inflated the scores on this self-report instrument. Nevertheless, it must also be kept in mind that the same motor and autonomic symptoms can mask anxiety symptoms during clinical evaluation.

The disentanglement of anxiety from motor symptoms in PD is both scientifically and clinically challenging [12], [17]. In our previous study [18], we found that the affective subscale was the only factor not associated with autonomic dysfunction, as measured with the Scales for Outcomes in Parkinson’s disease – Autonomic dysfunctions (SCOPA-AUT), and motor dysfunction as measured with the UPDRS-III. This is of clinical importance since it could indicate that certain items of the BAI should be weighted more heavily when screening for clinically relevant anxiety in PD. Therefore, in a post-hoc analysis, we investigated whether the score on the affective subscale of the BAI alone was better in predicting an anxiety disorder diagnosis given by a psychiatrist compared to the total score of the BAI. The AUC under the ROC curve was similar for both, indicating that the BAI total score and the BAI affective subscale score have similar predictive power. Using only the score on the affective subscale (7 items) in the prediction of an anxiety disorder diagnosis saves time and might be more practical compared to using the total BAI (21 items). In addition, this subscale might be considered as containing psychological and non-episodic anxiety items, which suspends the discussion about how to interpret the somatic BAI-items.

To evaluate the affective subscale further, comparison with other screening options for anxiety in PD is useful. The Movement Disorder Association currently does not recommend one specific anxiety screening instrument (latest published research from 2008 [27]), but does recommend the non-motor rating scale (the MDS-NMS) in which four questions about anxiety are included [28]. Two of those questions represent two items of the affective subscale of the BAI, the other two ask about panic attacks and social anxiety. Another screening tool for anxiety, the Parkinson Anxiety Scale (PAS), is a self-report questionnaire that includes items specifically for non-episodic anxiety and avoidance behavior [29]. The PAS excludes almost all somatic symptoms of anxiety, except for panic related symptoms (e.g. shortness of breath and heart palpitations). All non-episodic anxiety items of the PAS are comparable to the items that clustered together in the affective subscale.

This study has some limitations. The BAI focusses mostly on episodic anxiety, i.e. symptoms of panic disorder, while non-episodic anxiety, like in generalized anxiety disorder, is also common in PD. Unfortunately, autonomic dysfunction was not measured in the current patient sample, restricting the investigation of its associations with the (subscales of the) BAI. In terms of psychiatric diagnoses, this patient sample was not assessed with a structured clinical interview, to systematically check diagnostic criteria for all DSM diagnoses. Such structured clinical interviews, however, also risk overdiagnosis due to anxiety features being considered as part of a primary anxiety diagnosis, instead of secondary to another psychiatric diagnosis or PD-related symptoms of motor or autonomic failure, as can be expected in PD patients.

A major strength of this study is that we investigated a sample of PD patients who were assessed by two psychiatrists who are specialized in diagnosing anxiety in the context of motor and autonomic symptoms in PD patients. We successfully replicated the main findings by Rutten et al. (2015) [18], which makes these results more reliable and generalizable.

## 5 Conclusions

In this study, we replicated our previous findings of one affective and multiple somatic subscales of the BAI. The 7-item affective subscale of the BAI shows potential as a screening tool for non-episodic anxiety in PD. However, in clinical practice, it is recommended to evaluate anxiety symptoms in the context of other PD symptoms, including motor, autonomic, and other (neuro)psychiatric symptoms.

## Declaration of Competing Interest

The authors declare the following financial interests/personal relationships which may be considered: Odile A. van den Heuvel received speakers’ honorarium from BENECKE.
