# Supplementary material for: Symptom dimensions of anxiety in Parkinson’s disease: Replication study in a neuropsychiatric patient population
Source: Clin Park Relat Disord. 2021 Nov 10;5:100117. doi: 10.1016/j.prdoa.2021.100117 (PMC8605274; doi:10.1016/j.prdoa.2021.100117)
Supplement: Supplementary data 1 [file mmc1.docx]

**Table S1.** Matrix of the Pearson’s correlation coefficients (*r*) for the BAI, BDI, UPDRS-III, MoCA, and age.

|  | **BAI** | **BDI** | **UPDRS-III** | **MoCA** | **Age** |
| --- | --- | --- | --- | --- | --- |
| **BAI** | 1.000 | 0.599* | 0.015 | -0.248* | 0.123 |
| **BDI** | 0.599* | 1.000 | -0.44 | -0.141 | 0.27 |
| **UPDRS-III** | 0.015 | -0.44 | 1.000 | -0.319* | 0.306* |
| **MoCA** | -0.248* | -0.141 | -0.319* | 1.000 | -0.519* |
| **Age** | 0.123 | 0.27 | 0.306* | -0.519* | 1.000 |

*p-value < 0.01 (2-tailed)

BAI = Beck Anxiety Inventory; BDI = Beck Depression Inventory; UPDRS-III = Unified Parkinson’s Disease Rating Scale – part three (motor examination); MoCA = Montreal Cognitive Assessment.
