# Supplementary material for: Symptom dimensions of anxiety in Parkinson’s disease: Replication study in a neuropsychiatric patient population
Source: Clin Park Relat Disord. 2021 Nov 10;5:100117. doi: 10.1016/j.prdoa.2021.100117 (PMC8605274; doi:10.1016/j.prdoa.2021.100117)
Supplement: Supplementary data 2 [file mmc2.docx]

**Table S2.** Results of the unadjusted multiple linear regression analyses of the BAI total score and score on subscales of the BAI with the BDI, MOCA and UPDRS-III. Regression coefficients (B) with 95% confidence intervals (95% CI of B), standardized regression coefficients (β) and significance (indicated with*) are displayed.

| **DEPENDENT VARIABLE →** | ***Total BAI score*** | | ***Affective*** | | ***Thermoregulation*** | | ***Cardiopulmonary*** | | ***Unsteadiness*** | |
| --- | --- | --- | --- | --- | --- | --- | --- | --- | --- | --- |
| **INDEPENDENT**  **VARIABLE ↓** | B | 95% of B | B | 95% of B | B | 95% of B | B | 95% of B | B | 95% of B |
| BDI | 0.727* | 0.541 to 0.913 | 0.266* | 0.187 to 0.344 | 0.007 | -.039 to 0.052 | -.003 | -.040 to 0.035 | -.003 | -.073 to 0.067 |
| MoCA | -.597* | -1.044 to -.150 | -.252* | -.432 to -.072 | -.005 | -.096 to 0.086 | 0.000 | -.074 to 0.074 | 0.043 | -.096 to 0.182 |
| UPDRS-III | 0.012 | -.136 to 0.159 | -.014 | -.073 to 0.046 | 0.005 | -.024 to 0.034 | -.014 | -.037 to 0.010 | 0.003 | -.042 to 0.047 |

***** = p < 0.05
