# Supplementary material for: Symptom dimensions of anxiety in Parkinson’s disease: Replication study in a neuropsychiatric patient population
Source: Clin Park Relat Disord. 2021 Nov 10;5:100117. doi: 10.1016/j.prdoa.2021.100117 (PMC8605274; doi:10.1016/j.prdoa.2021.100117)
Supplement: Supplementary data 3 [file mmc3.docx]

| **Outcome measure** | **Mean (SD) Rutten et al. [18]** | **Mean (SD) current sample** | **Difference in mean (CI)** | **t-value** (df=415) | **p-value** |
| --- | --- | --- | --- | --- | --- |
| BAI | 14.2 (9.8) | 20.3 (11.4) | 6.1 (3.94 to 8.26) | 5.58 | <0.001 |
| BDI | 11.4 (8.0) | 18.2 (9.3) | 6.8 (5.03 to 8.57) | 7.63 | <0.001 |
| Age | 64.5 (10.3) | 66.1 (9.8) | 1.6 (-.53 to 3.73) | 1.48 | n.s. |
| UPDRS-III  (n=109) | 25.8 (12.3) | 27.3 (14.8) | 1.5 (-1.31 to 4.31) | 1.06 | n.s. |
| Disease duration (n=85) | 5.1 (5.6) | 8.4 (6.9) | 3.3 (1.94 to 4.66) | 4.81 | <0.001 |

**Table S3**. Independent samples t-tests results of the comparison of the sample characteristics between the current and the previous [18] patient sample.

BAI = Beck Anxiety Inventory; BDI = Beck Depression Inventory; UPDRS-III = Unified Parkinson’s Disease Rating Scale – part three (motor examination); MoCA = Montreal Cognitive Assessment, n = number of patients; SD = standard deviation; CI = Confidence Interval; df = degrees of freedom; n.s. = non-significant
